# Systematic Review and Meta-Analysis of Intervention Techniques in Occupational Therapy for Babies and Children with Obstetric Brachial Plexus Palsy

**DOI:** 10.3390/jcm13206186

**Published:** 2024-10-17

**Authors:** María Martínez-Carlón-Reina, Janine Hareau-Bonomi, Mª Pilar Rodríguez-Pérez, Elisabet Huertas-Hoyas

**Affiliations:** 1 Hospital Pediatric Sant Joan. Pg. de Sant Joan de Déu, 2, Esplugues de Llobregat, 08950 Barcelona, Spain; to.mamacare@gmail.com; 2 Head of Hand Therapy Clinic, Montevideo 11600, Uruguay; janine121958@gmail.com; 3 Physical Therapy, Occupational Therapy, Rehabilitation and Physical Medicine Department, Rey Juan Carlos University, 28922 Madrid, Spain; elisabet.huertas@urjc.es

**Keywords:** obstetric brachial plexus palsy, upper extremity dysfunction, occupational therapy, constraint-induced movement therapy, neuromuscular electrical stimulation, family education

## Abstract

**(1) Background**: Obstetric brachial plexus palsy (OBPP) is an unpredictable and unpreventable neurological injury, caused by shoulder dystocia during birth, that affects the brachial plexus and leads to motor and sensory deficits in the child’s upper extremity. The limited literature on early therapeutic assessment of newborns with OBPP highlights a gap in specialized care that, if filled, could enhance decision-making and support timely treatment. The objective of this paper is to analyze the therapeutic intervention techniques used at an early stage and their functional impact, from the occupational therapy discipline in the treatment of the upper extremity in babies and children with OBPP. **(2) Method**: Systematic review design and meta-analysis. A systematic review is a comprehensive analysis of existing research on a specific topic, using rigorous methods to identify, evaluate, and synthesize studies. Meta-analysis, often part of a systematic review, combines results from multiple studies to identify overall trends and enhance reliability, providing a clearer summary of evidence. Articles that included pediatric patients (from birth to 12 years of age) with a diagnosis of OBPP were reviewed. The results of the techniques used were analyzed according to each study, with the scale or method of assessment considered by the study for the presentation of data. The articles were assessed for methodological quality using the “PEDro Validity Scale”. **(3) Results**: A total of 2190 articles were found, with 108 analyzed and 22 fully meeting this study’s standards. Fourteen had a quantitative design, while the others included clinical guidelines. The most statistically reliable intervention techniques were CIMT (constraint-induced movement therapy) and splinting (dynamic and static), with second-tier techniques like joint manipulation, NMES, early infant management education, and serial casting used when needed. This study focused on children from birth to eight years old, with assessment tools primarily measuring upper limb range of motion, external rotation, supination, and impairment levels, though bimanual activity assessment was less common. **(4) Conclusions**: The early implementation of the techniques that provide us with the most data are CIMT, splinting, NMES, and joint manipulation linked to health education for families. In second place, we have the use of TB infiltrations and serial casts, when the treatment of the previous techniques fails in some cases.

## 1. Introduction

Obstetric brachial plexus palsy (OBPP) is a neurological and traumatic injury that occurs unpredictably and is unpreventable, commonly referred to as “shoulder dystocia”. It arises when the baby’s shoulders become impacted in the maternal pelvis, requiring additional maneuvers to complete the birth [1]. The incidence of obstetric brachial plexus palsy (OBPP) is estimated to range from 0.5 to 2.6 cases per 1000 live births, according to various epidemiological studies. This variability in incidence reflects differences in delivery protocols and access to early interventions. The majority of cases occur in the context of complicated vaginal deliveries, especially those associated with shoulder dystocia [2]. This condition affects the brachial plexus, impairing the function of the upper extremity and resulting in motor and sensory deficits in the child [3]. The severity and impact of the level or degree of obstetric trauma, as well as the significance and intensity of the consequences, will depend on the type and extent of the damage [4]. Due to the characteristics of the injury, OBPP often leads to unilateral muscle weakness, which can result in joint deformities in the upper extremity, among other issues, which significantly affect both family dynamics and the baby’s development [5]. Typical symptoms include a shoulder in adduction, internal rotation, an extended elbow without active movement, a dropped wrist in extension, and slightly flexed fingers. Additionally, infants may have difficulty rolling, manipulating their arms in space, and maintaining a prone position.

The diagnosis of OBPP is conducted by expert medical professionals (such as surgeons, specialized orthopedists, and rehabilitation physicians), using clinical examinations, diagnostic imaging, and other additional tests [5,6]. Medical diagnosis often relies on the Narakas classification and the Active Movement Scale, among other scales [6]. However, it is uncommon to find a complete multidisciplinary therapeutic team available in the early days of life of newborns suspected of having OBPP [3]. Additionally, electromyography (EMG) studies can provide crucial electrophysiological information to assess muscle activity and regeneration. In OBPP patients, EMG may reveal signs of denervation in affected muscles, which influences prognosis and treatment decisions. Diagnosis is further complemented by imaging studies such as magnetic resonance imaging (MRI) to evaluate structural damage to the brachial plexus [7].

The literature on early therapeutic assessment of newborns with OBPP is limited, highlighting a gap in specialized professional care that, if filled, could improve decision-making within the multidisciplinary team and facilitate appropriate early-stage treatment [3]. It is crucial to develop multidisciplinary clinical guidelines that adequately establish timing, evidence-based working techniques, and therapeutic protocols for early intervention to ensure the effective proper treatment of OBPP, leading to an improvement in the quality of life for affected children and their families [3].

The evidence highlights the necessity for early therapeutic supervision, as the first three months of life are critical for managing the condition and may prevent (though not always) the need for surgical intervention compared to patients who do not receive early care or who recover spontaneously. Early therapeutic treatment is essential to support the child’s development and empower families to actively participate in the care process [5].

Occupational therapy provides person-centered work focusing on individual needs. In cases of newborns with OBPP, occupational therapy empowers parents for a life journey with the child, addressing occupational challenges that arise from the condition, particularly those affecting the upper extremity during the early developmental stages [8].

This review aims to present the most evidence-based techniques within a multidisciplinary therapeutic approach, with a particular focus on the role of occupational therapists in the early assessment and treatment of OBPP from birth. Non-surgical, conservative treatment techniques with the strongest evidence can significantly impact the child’s outcomes [3].

The most effective therapeutic techniques during the first three months of life, with prior family education for daily and repetitive practice at home [3], include protecting and immobilizing the arm to prevent it from hanging, performing appropriate movements to maintain ranges of motion (particularly external and internal rotations), enhancing hand mobility, practicing elbow stimulation [9], promoting optimal positioning of the baby, and performing joint range manipulation and repeated passive mobilizations of the upper extremity several times a day [10]. Additionally, these techniques involve working on muscle tone and tissue elasticity [10] and utilizing sensory stimulation of the tactile and proprioceptive systems of the upper extremity and its functional use [9]. Occupational therapists may also recommend the use of dynamic and static splints or serial casting [1,11,12], and they may provide guidance on proper baby handling to support development [8]. After three months of age, controlled weight-bearing exercises to provide co-contraction to muscles and joints are recommended [10]. Other interventions include managing compensating movements, performing muscle strengthening using electrotherapy [13], using neuromuscular taping or Kinesio taping [12,14], and engaging the child in occupational activities that promote bilateral upper extremity use in daily life [6]. Additional techniques, such as neuromuscular electrical stimulation (NMES) [15], botulinum toxin (BT) injections [14,15,16], and constraint-induced movement therapy (CIMT) which involves restricting the unaffected limb during daily activities, have also shown efficacy [17,18].

In this meta-analysis, the objective is to analyze the effectiveness of early therapeutic intervention techniques focused on the upper extremity in children with OBPP from the perspective of occupational therapy. This study hypothesizes that early intervention with occupational therapy techniques in neonates with OBPP will significantly improve the range of motion and motor function of the affected upper extremity compared to patients who do not receive early treatment. Furthermore, it is expected that a multidisciplinary intervention integrating CIMT, dynamic and static splinting, and family education will contribute to faster and more sustained improvement over time. Through this analysis, we aim to provide new evidence on the importance of early intervention and personalized treatment in these patients.

## 2. Materials and Methods

This study employed a systematic review and meta-analysis design, following rigorous methods to identify, evaluate, and synthesize existing research on occupational therapy interventions for OBPP.

### 2.1. Eligibility Criteria

Inclusion criteria: Infants and children aged from birth to 12 years with a diagnosis of OBPP, studies addressing therapeutic treatment techniques in rehabilitation, and articles with relevant content about the injury, function, dysfunction, and intervention in the upper extremity from the occupational therapy approach were included. Exclusion criteria: Patients over 18 years of age, nerve impairments unrelated to obstetric injuries, articles with low methodological quality, articles over twenty years old, articles on surgical intervention techniques of any kind, and other systematic reviews or meta-analyses.

### 2.2. Search Strategy

Comprehensive searches were conducted in both paid and open-access scientific databases, including PubMed, Scopus, ScienceDirect, Web of Science, Cochrane, and SciELO, with a limit of up to 15 years from publication date.

### 2.3. Certainty Assessment

The “PEDro Validity Scale”, which provides a measurement tool for the quality of scales on a scale of 0–10, was used to assess the reliability of the articles. This scale provides a standardized method for measuring various dimensions of study quality, including the appropriateness of the research design, randomization, blinding, and outcome measures. By employing the PEDro scale, we ensured a rigorous assessment of methodological quality, which is crucial for determining the robustness of the evidence and minimizing bias. This systematic approach allows for a more reliable synthesis of the literature, ultimately enhancing the credibility of our findings and recommendations.

### 2.4. Data Extraction

A thorough review of the results presented in the selected articles was conducted to obtain data used in the systematic review and the meta-analysis. Data that could be compared were extracted from the studies for the systematic review, and a meta-analysis was conducted with those that were permitted by the characteristics of the studies.

### 2.5. Synthesis Methods

The study data correspond to the means represented in each study before and after the use of the treatment technique assessed. This was conducted by taking the mean reflected in the data of a study with respect to the assessment measure (PMAL, AHA, AMS, PROM, etc.) at the start of the study and after the clinical treatment period, obtaining the improvement in relative terms from baseline and in terms relative to the specific assessment scale used. The treatments included in the systematic review were CIMT, neuromuscular electrical stimulation (NMES), dynamic and static splinting, and joint manipulation techniques. These interventions were selected based on their documented effectiveness in improving upper limb function in children with OBPP.

The PEDro Validity Scale was used to assess the methodological quality of the included studies, ensuring that only studies with a score of 5 or higher were considered for the meta-analysis and synthesis.

A meta-analysis, which is a statistical technique that combines and summarizes the results of several individual studies, was performed. The study protocol was designed based on a comprehensive review of the existing literature on OBPP and the evaluation of the most effective therapeutic methods documented in previous clinical trials. Studies that utilized non-surgical techniques with strong evidence of functional improvement in children under 12 years of age were prioritized. The use of the PEDro scale ensured the methodological quality of the selected studies. The interventions included in the analysis were chosen based on their frequency of use and documented clinical efficacy, such as constraint-induced movement therapy (CIMT), dynamic and static splinting, and neuromuscular electrical stimulation (NMES).

## 3. Results

### 3.1. Study Selection

**Figure 1:**
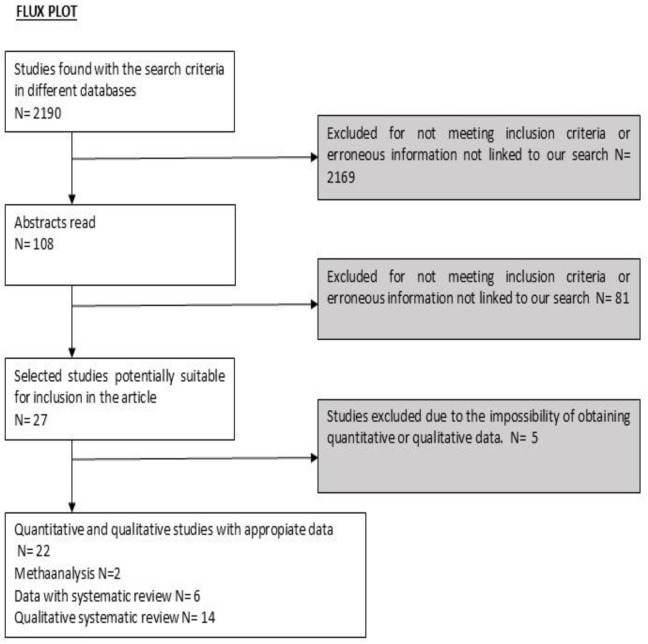
Flux plot: flux diagram for systematic review and meta-analysis.

A selection of 22 articles was made and included in this study, which contributed to its development. The results of the search can be seen in the flowchart (Figure 1) presented later in the results.

### 3.2. Study Characteristics

The results of methodological quality and study characteristics are shown in Table 1. There are only 17 articles because those with a validity score of less than five are not reflected, although they have been used as references as they represent clinical guidelines in the field.

**Figure 2:**
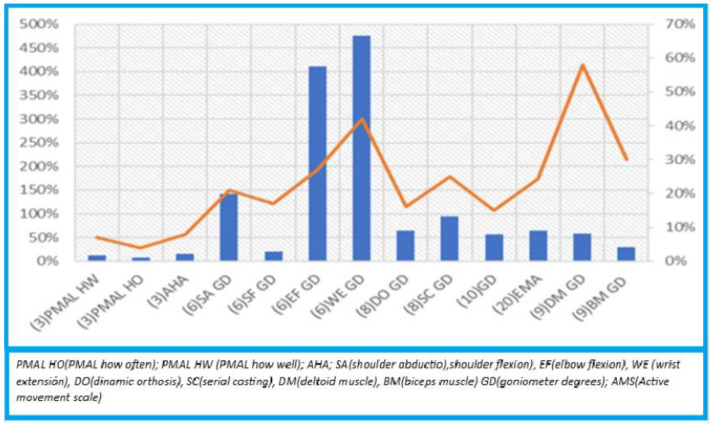
Improvement graph within scale range.

A meta-analysis was conducted, which is a statistical technique for combining and summarizing the results of several individual studies. In this case, studies [17,22] were used for the meta-analysis. The remaining six studies were evaluated based on the pre-and post-improvement of the specific treatment technique in each of them, as can be seen in Figure 2 in the Section 3.

First and foremost, as previously mentioned, it is important to reiterate that we have only achieved results for studies that we were able to apply a meta-analysis of [18,19], while for the remaining six [1,11,14,15,17,22], we conducted a quantitative data extraction to gauge the improvement offered by the evaluated treatment techniques in each study.

The selected studies for the meta-analysis were study [18] and study [19], as they were the only ones that met the necessary criteria, including both a control group and a study group, allowing for a comprehensive measurement of the analyzed effect.

The results of the meta-analysis have been established with a 95% confidence interval. Regarding heterogeneity, we used the I^2 indicator as a reference. Lastly, due to the limited number of different studies, which is two in this case, we obtained seven results through voluntary choice during the study.

To achieve broader coverage, we considered each of the results extracted from these two studies as individual studies. Study [18] provided five different results from the same sample, while study [19] contributed two results in both the control group and the study group, maintaining an equal sample size in both groups.

**Figure 3:**
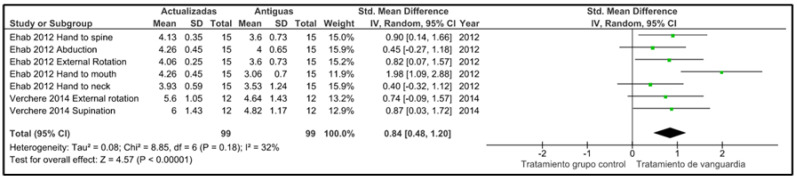
Forest plot [18,19].

It is important to note that when adjusting the data from two studies to obtain seven different results, the relevance of the overall effect observed in Figure 3 should be carefully considered.

In the analysis of the resulting forest plot, a favorable overall effect is observed for the updated treatments within the groups used as samples. However, it is important to note that both study [18] and study [17] present results with confidence intervals that cross the vertical line, indicating that these results do not reach statistical significance for the specific measure under consideration. Nevertheless, as mentioned previously, the overall effect conclusively demonstrates that the updated treatments are more effective compared to the older ones.

Regarding heterogeneity, which is common in this type of analysis, the I^2 statistic obtained was 33%, indicating a moderate degree of heterogeneity. Given that only two studies were available, these results suggest that there are sufficient differences between them and that caution should be exercised in interpretation. Furthermore, it is important to consider the disparity in the number of results obtained from each study, with five results from one study and only two from the other, introducing some asymmetry in their utilization.

Since most of the studies included in the systematic review are not reflected in the meta-analysis, it was not possible to perform a publication bias analysis in this context due to the limited number of available results.

Results of syntheses: During the data extraction process, we used a series of additional studies to analyze the results of updated treatments. Although these studies did not meet the necessary criteria for inclusion in the conducted meta-analysis, we decided not to discard the data we had extracted from them. Instead, we represented this data in a graph, referred to as Figure 3, which is presented later in the article.

In the graph, results from various measurements obtained from six of the studies included in our systematic review are presented. These studies yield different results for the evaluated treatments. For each of the results, we generated an improvement index relative to the starting point, based on the scale and table used to measure the average results of the groups in each study.

Let us take study [17] as an example. In this study, we have three results, two of which were assessed using PMAL (HOW WELL and HOW OFTEN), and the third was measured using the AHA scale. In addition to calculating the improvement relative to the starting point for each of the groups, we also applied a correction based on the magnitude of improvement within the scale’s range. This correction allows us to evaluate not only the improvement relative to the original point but also the improvement in absolute terms within the spectrum of possible results. By applying this, we aimed, to some extent, to compare the different results, as shown in Figure 3.

Following the same methodology, in the case of study [15], we have four results corresponding to different ranges of motion rehabilitated in the shoulder, elbow, and wrist. We used normal maximum ranges of motion as a reference for these results. When calculating the “improvement over the scale” indicator, we observed that although some improvements appear to be significant in relative terms, they are not as significant in absolute terms. Therefore, we applied the correction we designed to account for this discrepancy.

Regarding studies that include serial casting like [1,11], elbow flexion was measured in both cases. This allowed us to better compare the results, even with the specific elbow flexion result used in [15]. Since it is not exactly the same treatment and the sample size in all studies is not very large, some differences are observed, especially in terms of relative improvement. However, when considering improvement in relation to the scale used, the disparity in improvement is not as significant. It is important to note that multiple variables can influence each case, such as the age of the sample and the duration of treatment, so these differences may not necessarily have clinical significance.

Finally, we grouped the results of [22], which applied the EMA scale, and the results of [14] for the deltoid and biceps. The latter has the particularity that the improvement index coincides with the improvement in relation to the scale, as it represents a percentage of degenerated fibers.

## 4. Discussion

Within the results of the meta-analysis, we must identify two studies as relevant, as detailed above; these are [18], which is based on the implementation of the CIMT technique, and [19], which is based on the use of serial casts and static and dynamic splints. Both have control and study groups with an equal sample size; however, study [18] uses the Mallet Scale results to analyze improvement and exposes differences in at least five assessment categories (abduction; external rotation; hand to neck; hand to spine; hand to mouth), which is more detailed than study [19], which assesses through AMS and only presents two categories (supination and external rotation). It should be noted that having a greater number of assessment possibilities by evaluating five types of movements as opposed to two provides a more comprehensive analysis. Therefore, we believe that study [18] provides more relevant information regarding the capacity and ability of the upper limb, as assessed in this patient profile, compared to study [19]. Despite this, both studies show significant changes in comparing the results of control and study groups.

On the other hand, in relation to the systematic review, we were able to extract data from six studies, all of which showed improvement before and after the treatment provided by occupational therapy based on various assessment methods. In this context, it is worth highlighting that study [15], which involved parental education, joint manipulation techniques, and later focused on NMES, showed a very significant improvement compared to the others, with the starting point being 0 months. This emphasizes the importance of early treatment. Similarly, study [14], which used joint manipulation techniques and Kinesio taping, achieved very good results in absolute terms, with a median age of 2.5 months. Therefore, the best results overall were obtained in patient groups under 3 months of age, reinforcing the premise that the earlier the treatment is initiated, the more likely it is to yield better overall results with specific techniques trained and assessed by the occupational therapist. It should be noted that the use of NMES and Kinesio tape may be subject to disagreement among other authors and clinicians at such early ages.

Continuing in the same vein, we discuss study [17] with an average age of 25 months, which evaluates the use of CIMT, and study [22] with an average age of 33 months, which evaluates the use of TB. Obviously, the difference in results is not comparable in terms of parameters since different tests or types of assessment were used, and it should be highlighted that one technique complements the other according to other authors. It can be seen in Figure 3 that study [22] obtained significantly more improvement parameters overall, focusing on the assessment of the anatomical state of the upper limb musculature, while in study [17], which also showed significant results in terms of improvement percentage and overall improvement, these were analyzed through a functional scale, not just by specifying the anatomical part treated. For these reasons, we consider study [17] to be of greater interest.

Finally, we have study [11], which again deals with the serial casting technique in the elbow area, with an average age of 60 months, and study [1], which explores the options of serial casting and dynamic splints, with an average age of 128 months. Study [1] has a more obvious improvement in terms of overall improvement compared to study [11], which also shows overall improvement but to a lesser extent. Therefore, the combined use of both techniques, serial casting with dynamic splints [1], would be more effective than using one of them alone [11]. Furthermore, given the younger age of the subjects, they are more likely to improve according to the analyzed data.

The results obtained in the overall study were very limited, and we had to expand the inclusion and exclusion criteria to obtain a good method for analyzing and verifying with quantitative and qualitative data. In the same vein, we wanted to narrow down the age range to consider different studies, but it was not possible since there was not enough evidence for our study at early ages, with a maximum age of up to 3 years.

## 5. Conclusions

In conclusion, our study has highlighted the importance of early therapeutic intervention in children with OBPP from the perspective of occupational therapy. We have identified several effective techniques, such as CIMT, the use of dynamic and static splints, co-manipulation, and early infant management education with family involvement.

In terms of future research, we will work on investigating treatment techniques and assessment tools at an early age, with our goal being the development of these techniques for early treatment of newborns and their families. Finally, we consider the improvement of multidisciplinary communication, treatment timing, and family support, among other factors, highlighting the need for specific clinical guidelines developed by experts in the field and the implementation of these guidelines in our protocols.

## Figures and Tables

**Table 1:** Study characteristics and methodological quality (author, mean age, N, intervention, treatment duration, measurement scale, and methodological quality).

Author/Year	Mean Age	(N)	Intervention	Treatment Span	Measurement Scale	Methodological Quality on PEDro Scale (0 to 10)
Delioglu K. 2022 [5]	56 months	112	Preschool manipulative skills	8 months	Brachial plexus outcome measure (BPOM)	9
Ehab M.A-K. 2013 [18]	50 months	15	CIMT	12 weeks	Mallet, Goniometer	8
Werner J.M. 2020 [17]	25 months	21	CIMT	8 weeks	Ability Hand Assisting (AHA)	10
Justice D. 2018 [15]	0 months	11	Neuromuscular electrical stimulation and other complementary rehabilitation techniques	6 weeks	Active Range of Motion (AROM)	7
De Coul OP L.S. 2023 [1]	128 months	55	Dynamic splints vs. Serial casting	12 months	Medical Research Council (MRC)	10
Elkhatib R.S. 2012 [15]	2.5 months	30	Kinesio taping in rehabilitation treatments of occupational therapy and physiotherapy	3 months	Active Motion Scale (AMS)	6
Duijnisvelda B.J. 2016 [11]	60 months	20	Serial casting	4–6 weeks	Medical Research Council (MRC)	8
Verchere C. 2014 [19]	10 months	12	Supination and external rotation splint	2.5 months	Active Movement Scale (AMC)	10
Buitenhuis. 2023 [9]	120 months	41	Not specified	None	Semmes–Weinstein monofilament	8
Pons C. 2019 [16]	10 months	62	Botulinum toxin (internal rotators)	15 months	Magnetic resonance imaging	8
Moscher A.M. 2020 [20]	78 months	12	Botulinum toxin (triceps)	8 months	The Toronto test score	7
Heise O.C. 2005 [21]	24 months	8	Botulinum toxin (triceps and biceps)	18 months	Electromyography and Medical Research Council (MRC)	7
Garcia Ron A. 2017 [22]	33 months	15	Conservative clinic treatment	4 years	Active Movement Scale (AMS)	4
Manske C.M. [23]	0 months	66	Not specified	3 months	Study-specific questionnaire	9

## Data Availability

The data presented in this study are available on request from the corresponding author.
